# Lowering the n-6/n-3 PUFAs ratio inhibits the formation of THP-1 macrophage-derived foam cell

**DOI:** 10.1186/s12944-018-0772-y

**Published:** 2018-05-25

**Authors:** Zhixiu Song, Hui Xia, Ligang Yang, Shaokang Wang, Guiju Sun

**Affiliations:** 1 Second Clinical Medical College, Nanjing University of Chinese Medicine, 138 Xian Lin Road, Nanjing, 210046 China; 2 Key Laboratory of Environmental Medicine and Engineering of Ministry of Education, and Department of Nutrition and Food Hygiene, School of Public Health, Southeast University, 87 Ding Jia Qiao Road, Nanjing, 210009 China

**Keywords:** The n-6/n-3 PUFAs ratio, Atherosclerosis, Foam cell, Inflammation, Cholesterol homeostasis

## Abstract

**Background:**

The balance between n-6 and n-3 PUFAs is an important determinant in the risk for cardiovascular disease. The study was to investigate the influence of the n-6 and n-3 PUFAs ratio on the formation of THP-1 monocyte-derived foam cells and explore the probable mechanism of anti-atherosclerosis.

**Methods:**

THP-1 monocyte cells were cultured with PMA and ox-LDL to establish a foam-cell model, while treated with different ratios of n-6 to n-3 PUFAs for 48 h. The cholesterol of foam cells was measured by a cholesterol assay kit. The levels of IL-6 and TNFα in supernatant were detected with ELISA methods. The expressions of CD36, ABCA1, ACAT1, PPARγ and LXRα mRNA were detected with real-time PCR.

**Results:**

Compared with the foam cell model group, the low and middle ratio of n-6 to n-3 PUFAs groups decreased the intracellular concentration of cholesterol (*P* < 0.01), but the high n-6/n-3 PUFAs ratio did not. Fatty acids decreased the level of IL-6 and TNFα in supernatant in a ratio-dependent manner. Fatty acids treatment also decreased the expressions of CD36、ACTA1、PPARγ、LXRα mRNA in a ratio-dependent manner.

**Conclusions:**

Lowering the ratios of n-6 to n-3 PUFAs can decrease the secretion of inflammatory cytokines then reduce the expressions of CD36 and ACAT1 mRNA. As well, it can decrease the expressions of CD36 mRNA through the PPARγ pathway. This leads to less cholesterol ingestion into the cells and decreased synthesis of cholesteryl ester, which inhibits the formation of the foam cells, further preventing the occurrence and development of atherosclerosis.

## Background

Polyunsaturated fatty acids (PUFAs) are important fatty acids, including n-6 and n-3 PUFAs according to the location of the double bond. n-6 PUFAs are known to compete with n-3 PUFAs in the metabolic pathways as they share the same series of enzymes such as desaturase- and elongase-enzymes etc. [1, 2]. Arachidonic acid (AA) and eicosapentaenoic acid (EPA) are representatives of n-6 PUFAs and n-3 PUFAs respectively, because they are the precursors for the production of eicosanoids. But eicosanoids from AA are pro-inflammatory, whereas those from EPA are anti-inflammatory [2, 3]. The present diet is deficient in n-3 fatty acids with a ratio of n-6 to n-3 of about 10:1, instead of 1:1 that was during evolution in humans [4]. And the recommended nutrient intake for n-6/n-3 ratio is 4-6:1 in China [5]. Numerous epidemiological and clinical studies have shown that an unbalance of n-6 and n-3 promote the pathogenesis of many disease, including cancer, cardiovascular disorders, asthma depression and autoimmune disorder [4, 6].The possible mechanisms that may contribute to the cardiovascular benefits of n-3 PUFAs is their ability to improve the lipid metabolism and reduced synthesis of inflammatory eicosanoids from n-6 PUFAs. Thus the balance between n-6 and n-3 PUFA is an important determinant in decreasing the risk for cardiovascular disease and in the prevention of atherosclerosis.

Atherosclerosis is not only a lipid disorder, but also a chronic inflammatory disease [7–9]. It has been accepted that inflammation plays an important role in both the initiation and progression of atherosclerosis. The different ratios of n-6 and n-3 PUFAs have different effects on lipid metabolism and inflammatory response, so the ratio is associated with the initiation and development of atherosclerosis. Despite of the complicated factors in the initiation and development of atherosclerosis, the central hallmark is the formation of the foam cell [10]. The cholesterol homeostasis in macrophages is a determining factor towards the formation of foam cells, which includes increasing of cholesterol uptake and biosynthesis, decreasing of cholesterol efflux. Most studies concentrate in the effect of the single kind of fatty acids on the cholesterol homeostasis and the formation of the foam cell, but not much is known about the effect of n-6 / n-3 PUFAs ratio. The objective of this study is to investigate the influence of the n-6 PUFAs (AA) and n-3 PUFAs (EPA) ratio on the formation of THP-1 monocyte-derived foam cells and explore the probable mechanism of anti-atherosclerosis.

## Method

### Materials

EPA and AA, dimethyl sulfoxide (DMSO), 3-(4, 5-dimethyl-thiazol-2-y)-2.5-diphenyl tetrazolium bromide (MTT), Phorbol-12-myristate-13-acetate(PMA) and Oil Red O were obtained from Sigma–Aldrich USA. Phosphate buffered saline (PBS) tablets were provided by Takara, Japan. Fetal bovine serum (FBS) were obtained from Invitrogen Corporation. Ox-LDL was purchased from YiYuan Biotech (Guangzhou, China). Fatty acids free BSA was obtained from Equitech-bio company (US). The ELISA kits of IL-6 and TNFα was purchased from Science Biotechnology Co. Ltd. (Yantai, China). The cholesterol assay kit was purchased from Applygen Technologies Inc. (Beijing, China). The protein assay kit was purchased from Thermo Fisher Scientific (Rockford, IL). Reagents and kits for RNA extraction and reverse transcription were obtained from Qiagen, USA.

### Foam cell formation

Human THP-1 monocytes cell lines purchased from Shanghai Cellular Institute of China Scientific Academy were used for these studies. Cells were cultured in RPMI 1640 culture medium with 10% fetal bovine serum (FBS), 10 nM L-glutamine, and a mixture of antibiotics (penicillin and streptomycin) in a humid atmosphere containing 5% CO2 at 37 °C. After induced by 160 nmol/L PMA for 48 h, THP-1 monocyte cells differentiated into macrophages. Then macrophages were transformed to foam cells by incubation with 50 μg/mL ox-LDL for 48 h. Cells were stained with Oil Red O and rinsed in 60% isopropanol and water then multiple images were taken.

### Fatty acid preparation

EPA or AA was prepared in ethanol (the final concentration was ≤0.2%) and diluted with RPMI 1640 culture medium supplemented with 0.5% BSA. In order to obtain the desired final concentrations of EPA and AA, MTT assays were used to evaluate cell viabilities. The desired final concentrations of EPA and AA were mixed together to obtain the specific ratio of 1, 5 and 10, these were defined as low ratio, medium ratio and high ratio respectively.

THP-1 monocytes incubated with 50 μg/mL ox-LDL in the presence of fatty acids with different ratio of AA to EPA (Low ratio group, Medium ratio group and High ratio group). And THP-1 monocytes incubated with 50 μg/mL ox-LDL was defined as the model group and without ox-LDL as the control group.

### Lipid extraction analysis

After treated with fatty acids for 48 h, the cells were washed in PBS for three times. The cellular lipids were extracted with lysis buffer, the mixture was then centrifuged (2000 g, 10 min), and the supernatant was used to determine the cholesterol level with an enzymatic analysis by cholesterol assay kit. Protein concentrations were also quantified. The cholesterol concentration was expressed as nmol/mg protein.

### Detection of soluble cytokines in culture supernatants

THP-1 macrophages were treated with fatty acids for 48 h, then culture supernatants were collected for measurement of cytokine (IL-6 and TNFα) production. All cytokines were measured using ELISA kits according to the manufacturer’s instructions.

### RNA extraction and quantitative RT-PCR

Total RNA was isolated using Trizol Reagent according to the manufacturer’s recommended protocol. The RNA purity and concentration was determined by eppendorf Biophotometer and RNA quality was identified using electrophoresis. Two micrograms total RNA was used as template to synthesize cDNA with M-MLV Reverse Transcriptase kit. Real-time PCR was started by heating at 94°Cfor 5 min, followed by 35 cycles at 94 °C for 30s, 56°Cfor 30s, and 72 °C for 45 s, with a final extension at 72 °C for 5 min. Quantification was normalized to the amount of endogenous GAPDH. Forward and reverse primer sequences are shown in Table 1.

**Table 1:** Primer sequences of PCR reaction

Gene		sequences	lengths
GAPDH	Forward	5’-CACCCACTCCTCCACCTTTG-3’	110 bps
	Reverse	5’-CCACCACCCTGTTGCTGTAG-3’	
CD36	Forward	5’-AGTTGGAGACCTGCTTATC-3’	163 bps
	Reverse	5’-GTTGCTGCTGTTCATCATC-3′	
ABCA1	Forward	5’-TAACATCCTGCTTTGATTCC-3’	193 bps
	Reverse	5’-TTGATTCTTTGCCTCTTACC-3’	
ACAT1	Forward	5’-TGAAGGAAGTGGTCATAGTAAG-3’	246 bps
	Reverse	5’-TGGTACATGGAGTAGAAATAGG-3’	
LXRα	Forward	5’-TCGGGCTTCCACTACAATGTTC-3’	234 bps
	Reverse	5’-CTCTTGCCGCTTCAGTTTCTTC-3’	
PPARγ	Forward	5’-TCCACGAGATCATTTACAC-3’	155 bps
	Reverse	5’-CTTCACAGCAAACTCAAAC-3’	

### Statistical analysis

All experiments were carried out in triplicate and the results were expressed as mean ± S.D. The statistical difference between groups was determined by one-way ANOVA followed by Dunnett’s post hoc test using SPSS 17.0 software. *P* < 0.01 was considered statistically significant.

## Results

### Macrophage foam cell formation

**Fig. 1:**
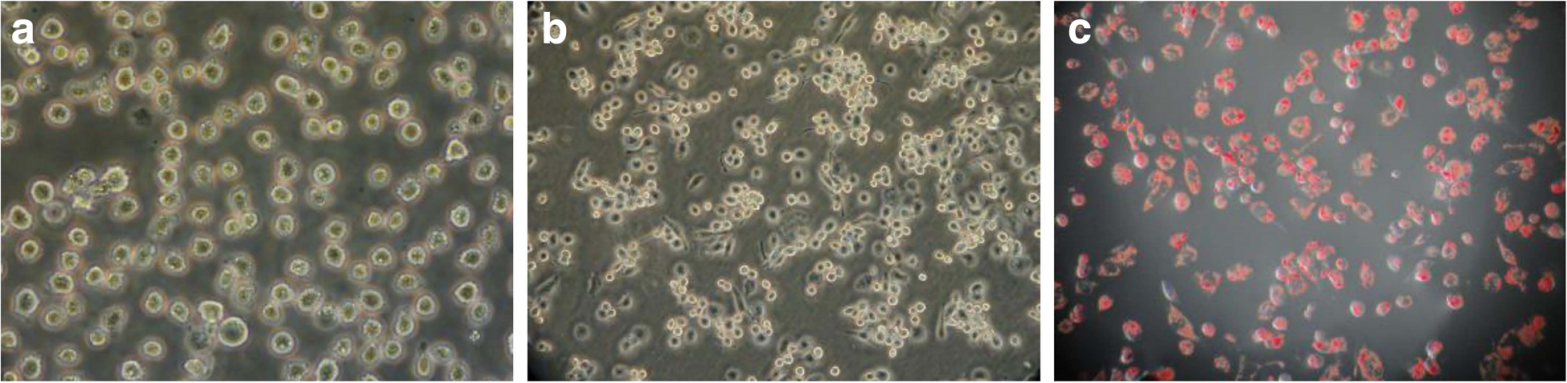
Transformation of macrophages to foam cells.THP-1 monocytes (**a**), THP-1 derived macrophages (**b**) and foam cell identified by Oil Red O staining (**c**)

In the presence of PMA, most THP-1 cells attached to the bottom of dish and differentiated into macrophages (Fig. 1a and b). Following ox-LDL treatment, the THP-1–derived macrophages took up lipids and differentiated into foam cells (Fig. 1c). Red oil O staining confirmed the formation of foam cell while large amounts of oil red O which stained lipid droplets were visible.

### Identification of the optimum incubation concentration of AA and EPA

**Fig. 2:**
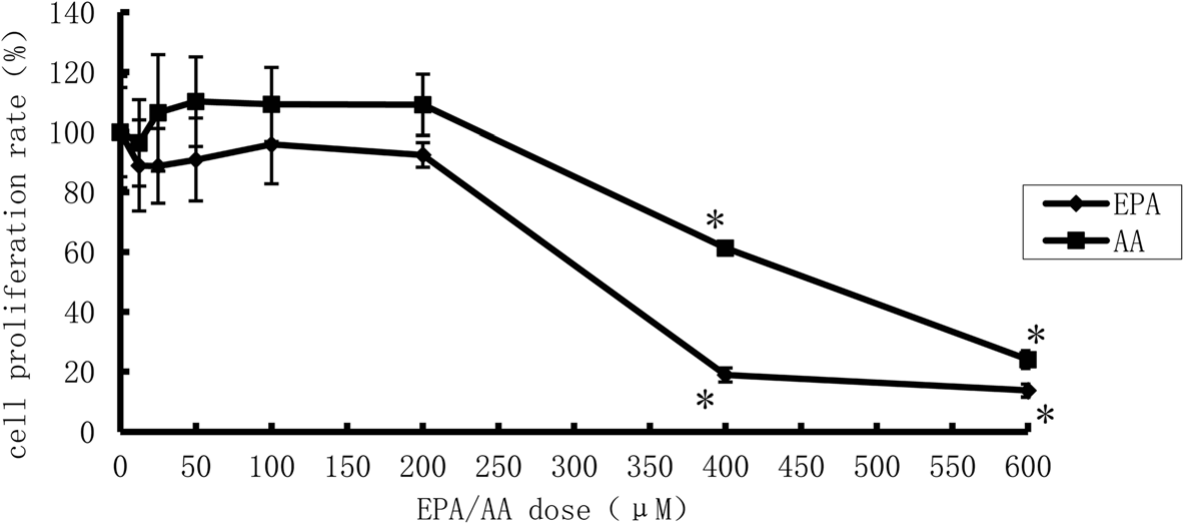
Cell proliferation rate after EPA or ARA treatment (*n* = 5).* vs EPA or ARA =0 μM, *P*< 0.01

The THP-1-derived macrophages were treated for 48 h by EPA or AA with dose gradients. Compared with the control group, 48 h of incubation with AA or EPA with doses ranging from 12.5 to 200 μM had no effect on macrophage viability, and at least 85% cells survived in each group (Fig. 2). However, when the concentration was elevated to 400 μM, the FFAs markedly decreased cell viability (*P* < 0.01) separately. Therefore, we chose 100 μM as the optimum concentration for macrophages incubation in the following study.

### Lower ratio of AA and EPA inhibited lipid accumulation in foam cells

**Fig. 3:**
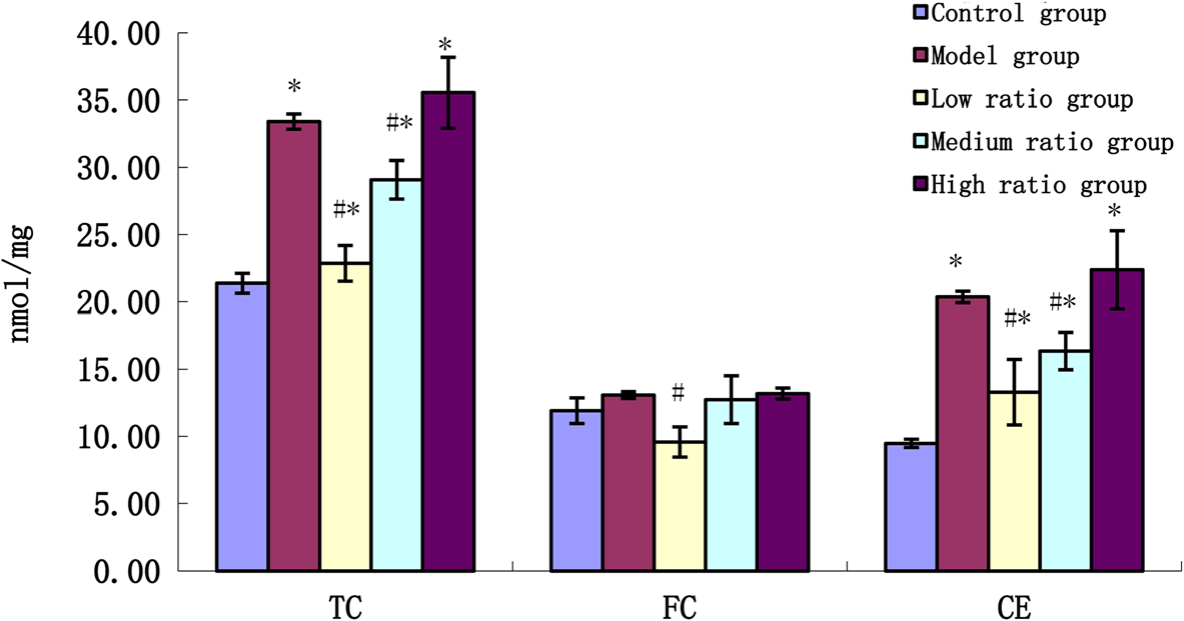
The effects of n-6/n-3PUFAs ratio on the concentration of cholesterol in foam cells. * vs control group, *P*<0.01; # vs model group, *P*<0.01

To investigate the effects of different ratios of AA and EPA on lipid accumulation during THP-1-derived macrophage foam cells formation, we measured intracellular total cholesterol (TC), free cholesterol (FC) and CE levels in foam cells. As presented in Fig. 3, incubation with low and medium ratios caused a significant decrease in intracellular TC and CE concentrations (*P* < 0.01), and the low ratio FFAs also caused a significant decrease in FC. High ratio of AA/EPA could elevate the level of TC, FC and CE, but it was not significant (*P* = 0.117; *P* = 0.807; *P* = 0.206, respectively). These results indicated that the low and medium ratios of AA/EPA could inhibit the formation of the foam cell, but not the high ratio. Compared to the control group, incubation with different ratios of AA/EPA caused a significant increase in CE concentrations (*P* < 0.01), and the level of TC increased in the medium and high groups (*P* < 0.01).

### PUFAs inhibit the secretion of the inflammatory cytokines in foam cells

**Fig. 4:**
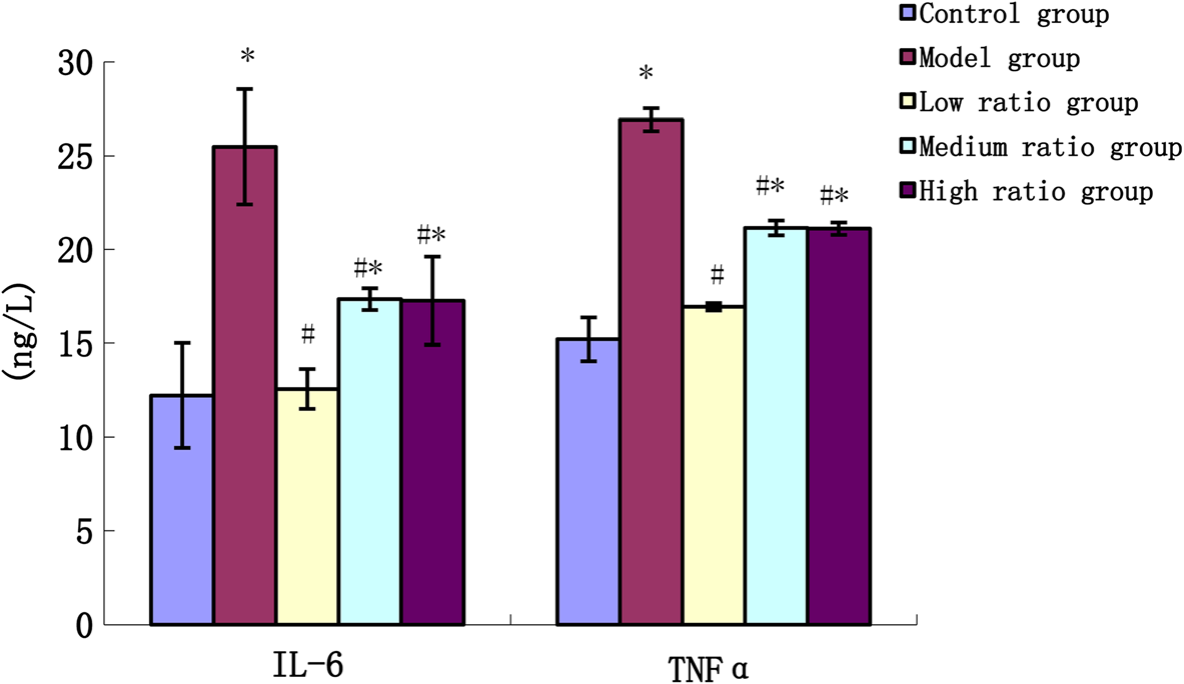
The effects of n-6/n-3 PUFAs on the content of inflammatory cytokines in supernatant of culture solution. * vs control group, *P*<0.01;# vs model group, *P*<0.01

As shown in Fig. 4, the level of the inflammatory cytokines (IL-6 and TNFα) in the model group increased significantly compared to the control group (*P* < 0.01), and incubation with different ratios of AA/EPA caused a significant decrease in IL-6 and TNFα concentrations (*P* < 0.01) compared to the model group. Compared to the control group, the level of the inflammatory cytokines (IL-6 and TNFα) in the medium and high ratio groups increased significantly (*P* < 0.01).

### Assessment of gene expression of CD36, ABCA1, ACAT1

**Fig. 5:**
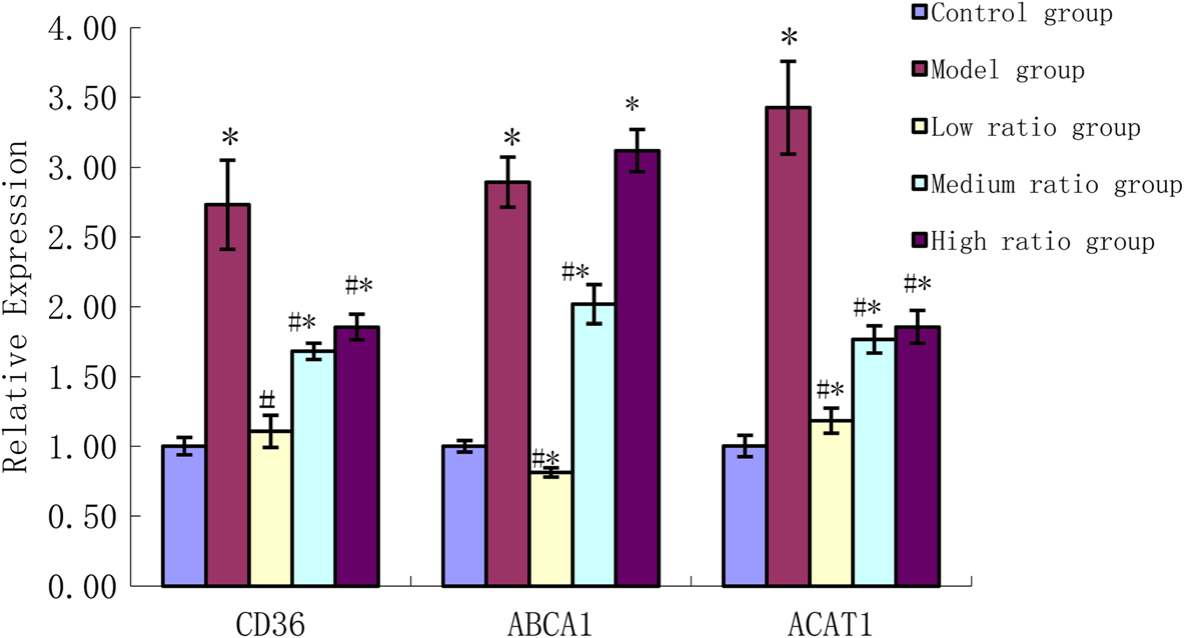
The effects of n-6/n-3 PUFAs on the expressions of CD36, ABCA1 and ACAT1 mRNA. * vs control group, *P*<0.01;# vs model group, *P*<0.01

As shown in Fig. 5, the mRNA expression of a member of the type B family (CD36), ATP-binding membrane cassette transport protein A1 (ABCA1), Acyl-Co A: Cholesterol Acyltransferase (ACAT1) in the model group increased significantly compared to the control group. Compared to the model group, PUFAs were observed to significantly downregulate the mRNA expression of CD36 and ACAT1 (*P* < 0.01). Treatment with the low and medium ratios of AA/EPA decreased ABCA1 mRNA levels (*P* < 0.01), while the high ratio increased ABCA1 mRNA levels but did not show significant effect. Compared to the control group, the mRNA expression of CD36, ABCA1, ACAT1 in the medium and high ratio groups increased significantly (*P* < 0.01), but the mRNA expression of ABCA1 in the low ratio group decreased significantly (*P* < 0.01).

### Assessment of gene expression of PPARγ, LXRα

**Fig. 6:**
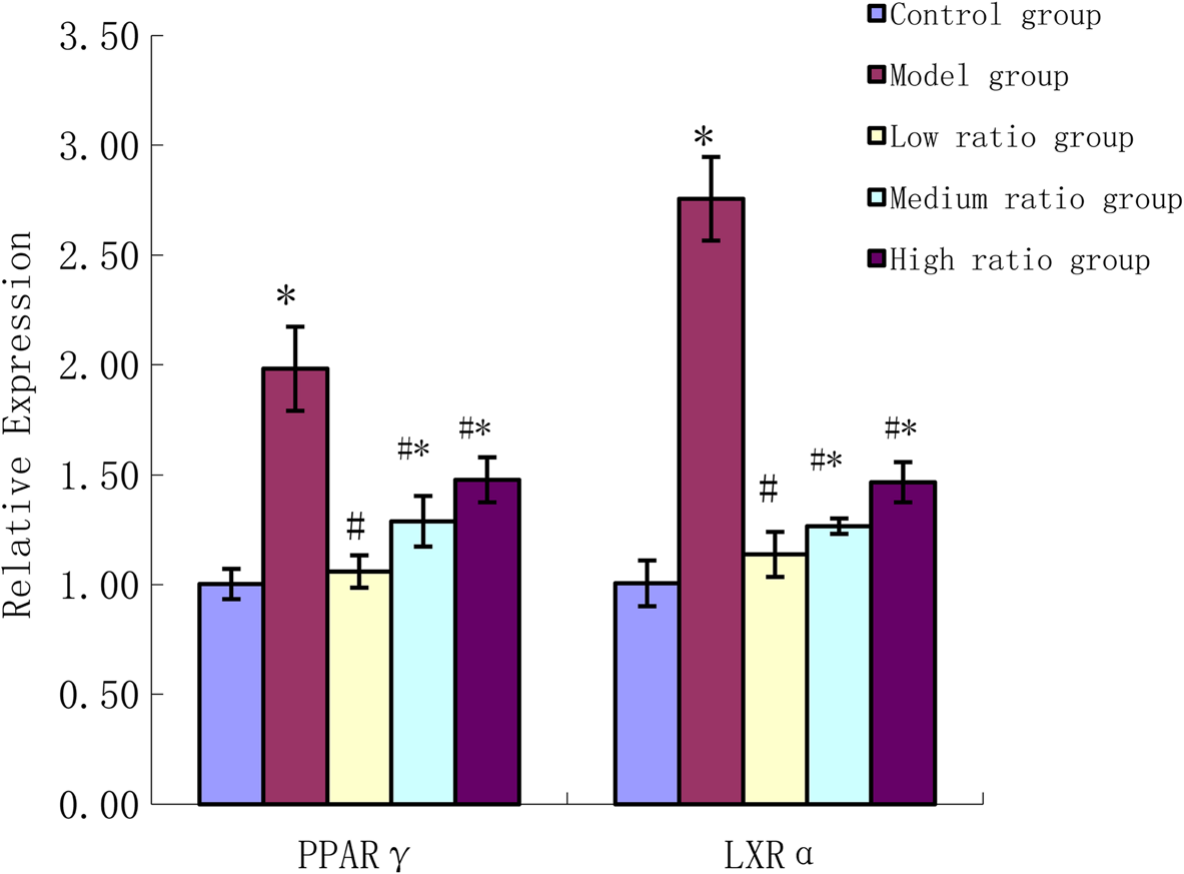
The effects of n-6/n-3 PUFAs on the expressions of PPARγ、LXRαmRNA. * vs control group, *P*<0.01;# vs model group, *P*<0.01

As shown in Fig. 6, the mRNA expression of peroxisome proliferator-activated receptor γ (PPARγ), liver X receptor α (LXRα) in the model group increased significantly compared to the control group. Compared to model group, PUFAs were observed to significantly downregulate the mRNA expression of PPARγ, LXRα (*P* < 0.01). Compared to the control group the mRNA expression of PPARγ, LXRα in the medium and high ratio groups increased significantly (*P* < 0.01).

## Discussion

The formation of foam cells by macrophages in the intima play a critical role in the occurrence and development of atherosclerosis, so inhibiting lipid overload within macrophages and formation of foam cells may be a valuable therapeutic method in preventing coronary artery disease [11, 12]. Uncontrolled uptake of oxidized low-density lipoprotein (ox-LDL) by macrophages result in accumulation of both CE and triglyceride (TG) stored as cytoplasmic lipid droplets and subsequently trigger the formation of foam cells [13]. This present investigation illustrated that after treatment with ox-LDL for 4 h the macrophages showed the morphological and biochemical characteristics of foam cells, thus indicating the formation of the foam cells. In the presence of fatty acids with different ratios of AA and EPA, the medium and low ratio groups decreased the accumulation of the TC and CE, while the high ratio group did not and in contrast the content of TC and CE was higher than that of the model group. Previous studies have demonstrated that macrophage pretreated with NEFA could increase lipid content, but LA and EPA showed positive results in lower lipid content than oleic acid (OA) and palmitic acid (PA) [14]. This suggests that different kinds and composition of fatty acids have different functions in foam cells and the ratio of n-6 and n-3 also has an important influence on the formation of foam cells.

Atherosclerosis is a chronic inflammatory proliferative disease. In the course of macrophage-derived foam cell formation, a lot of inflammatory factors are released just induced by ox-LDL [15]. Among these inflammatory factors, IL-6 and TNFα play an important role [16]. IL-6 and TNFα are produced chiefly by activated macrophages, and they are known to influence lipid metabolism and regulate the synthesis of other acute phase proteins which are established risk factors for atherosclerosis [17]. In this study, we found that incubation of THP-1 derived macrophages with 50 μg/mL ox-LDL can significantly increase the concentration of IL-6 and TNFα. After an intervention with different ratios of fatty acids, the concentration of IL-6 and TNFα declined to different degrees, with the low ratio group showing the most reduction. A previous study demonstrated that EPA at 100 μM concentration effectively suppressed the production of IL-6 and TNFα in LPS and PGE2 stimulated macrophages, but this effect was not as dramatic when EPA was used as a mixture with AA [18]. The anti-inflammatory potential of n-3 is not only due to the reduced synthesis of inflammatory eicosanoids from n-6 PUFAs, but also the selective activation of leukocyte GPR120/FFAR4 by n-3 PUFAs [19]. It can be concluded that n-3 fatty acids can suppresses the inflammatory response, and this may be another pathway of inhibiting the formation of the foam cell via reduced ratio of n-6 and n-3 PUFAs. In this study it should be mentioned that anti-inflammatory effects of all ratios are similar but cholesterol lowering ability is only preserved in the low n-6/n-3 ratio, which could be due to reduced de-novo lipogenesis by n-3 PUFAs through sterol-regulatory element binding proteins 1 (SREBP1) pathway in vivo [20].

It has been well known that modified forms of LDL are taken up by the scavenger receptors with extremely high efficiency and this can lead to massive lipid accumulation and foam cell formation. Therefore, the upregulation of the scavenger receptors can increase the intake of cholesterol. CD36 is the major receptor for the uptake of ox-LDL [21], and macrophages derived from CD36-apoE double-null mice bounded and internalized less ox-LDL as compared with apoE-null macrophages [22]. The role of CD36 is to recognize and internalize ox-LDL and can also be upregulated by ox-LDL, which is shown to be necessary for the differentiation of macrophages into foam cells [23]. In this study the mRNA expression of CD36 in the model group has increased significantly and fatty acids can modulate CD36 expression. We found that PUFAs inhibited the mRNA expression of CD36, especially in the low ratio group. Pietsch etc. also found that n-3 fatty acids inhibited CD36 expression while n-6 fatty acids tended to increase expression of CD36 [24]. This suggests that decreasing the ratio of n-6 to n-3 can downregulate the expression of CD36 and decrease the ingestion of cholesterol, which in turn prevents the formation of macrophage foam cells.

In macrophages, the accumulation of cholesteryl esters synthesized by the activated acyl-coenzyme A:cholesterol acyltransferase-1 (ACAT1) results in the foam cell formation, which is a hallmark of early atherosclerotic lesions. We found that the mRNA expression of ACAT1 was increased 3.43 times in THP-1-derived macrophage foam cells, whilst there was an accumulation of cholesteryl esters from 9.43 ± 0.37 nmol/mg to 20.35 ± 0.42 nmol/mg. PUFA downregulated the mRNA expression of ACAT1, especially in low ratio group. These data support the hypothesis that low ratio of n-6 and n-3 PUFAs inhibiting the formation of the foam cell may in part be due to weakening the ACAT1 expression to alleviate the accumulation of cholesteryl esters.

Low ratio of n-6 to n-3 can reduce cholesterol uptake and biosynthesis, but it is unknown whether low ratios can strengthen the cholesterol efflux via reverse cholesterol transport. As a cholesterol efflux regulatory protein, ABCA1 can promote cholesterol efflux in an indirect fashion. ABCA1 Functions as a cholesterol efflux regulatory protein [25]. Ox-LDL upregulates expression of ABCA1 in THP-1-derived macrophages and enhances the cholesterol efflux, and also decrease cholesterol uptake and biosynthesis, but to a lesser extent [26, 27]. In our study, after THP1-derived macrophages were incubated with ox-LDL (50 mg/L), the mRNA expression of ABCA1 was increased 2.89 times, but PUFA downregulated the mRNA expression of ABCA1. PUFAs may not be able to accumulate enough lipids to upregulate the gene related with the cholesterol efflux, however, PUFAs can enhance ABCA1 degradation [28]. This demonstrates that low ratio of n-6 to n-3 inhibits the formation of the foam cells as the quantity of cholesterol uptake via CD36 and biosynthesis via ACAT1 is less than the cholesterol efflux via ABCA1, which co-regulate to inhibit the development of the atherosclerosis.

As a member of a nuclear hormone superfamily, PPARγ plays essential roles in the occurrence and development of atherosclerosis, which might be due to its regulation of glycolipid metabolism, involvement in inflammation and activation of cholesterol efflux/reverse cholesterol transport [29]. Activation of PPARγ can promote uptake of ox-LDL through induction of CD36 gene expression [30, 31]. In addition to promoting lipid uptake, PPARγ induces ABCA1 expression and cholesterol removal from macrophages by positive regulation of the Liver X Receptor (LXR) gene [32, 33]. The secretion of inflammatory cytokines, such as TNFα and IL-6 can promote lipid uptake and biosynthesis by inducing the expression of CD36 and ACAT1. In this work, we found that uptake of ox-LDL by macrophages resulted in induction of PPARγ, and then induction of CD36 and ABCA1 through LXR. PPARγ coupled a pathway of ox-LDL uptake to a pathway of cholesterol efflux. Maybe in macrophages the quality of cholesterol uptake and biosynthesis is more than cholesterol efflux. So the level of TC and CE in the model group was higher than that in the control group. PUFAs especially with low n-6/n-3 PUFAs ratio can inhibit the formation of foam cells, mainly due to PUFAs inhibiting CD36 expression and cellular ox-LDL accumulation by inactivating the PPARγ pathway. In addition, PUFAs with low n-6/n-3 PUFAs ratio could also reduce the secretion of inflammatory cytokines and downregulate the expression of CD36 and ACAT1. Hence, inhibiting the production of inflammatory cytokines can be attributed to the anti-atherogenic properties of PUFAs with low n-6/n-3 PUFAs ratios.

## Conclusions

Decreasing the ratio of n-6 to n-3 PUFAs can downregulate the expression of ABCA1 mRNA through PPARγ/LXRα/ABCA1 pathway, and inhibit cholesterol efflux. It can decrease the secretion of inflammatory cytokines then reduce the expressions of CD36 and ACAT1 mRNA. As well, it can decrease the expressions of CD36 mRNA through the PPARγ pathway. The ability of PUFAs when there is a greater amount of n-3 and lower amount of n-6 resulting in a lower ratio of n-6 to n-3 is shown to inhibit the cholesterol ingestion into the cell and synthesis of cholesteryl ester is stronger compared to the inhibition of the cholesterol efflux. So lowering n-6/n-3 PUFAs can inhibit the formation of the foam cell, further preventing the occurrence and development of atherosclerosis.

## Abbreviations

AA: Arachidonic acid
ABCA1: ATP-binding membrane cassette transport protein A1
ABCG1: ATP-binding membrane cassette transport protein G1
ACAT1: Acyl-Co A: Cholesterol Acyltransferase
BSA: Bovine serum albumin
CD36: Type B family
CE: Cholesteryl ester
DMSO: Dimethyl sulfoxide
EPA: Eicosapentaenoic acid
FBS: Fetal bovine serum
FC: Free cholesterol
LXRα: Liver X receptor α
MTT: 3-(4, 5-dimethyl-thiazol-2-y)-2.5-diphenyl tetrazolium bromide
OA: Oleic acid
ox-LDL: Oxidized low-density lipoprotein
PA: Palmitic acid
PBS: Phosphate buffered saline
PMA: Phorbol-12-myristate-13-acetate
PPARγ: Peroxisome proliferator-activated receptor γ
PUFA: Polyunsaturated fatty acids
SRA: Type A scavenger receptor
SREBP1: Sterol-regulatory element binding proteins 1
TC: Total cholesterol
TG: Triglyceride
